# The puzzle of pain and its psychological implications: Differentiating complex regional pain syndrome from somatic symptom disorder

**DOI:** 10.1371/journal.pmen.0000214

**Published:** 2025-01-02

**Authors:** Paul D. C. Zimmer, Robert T. Rubin

**Affiliations:** 1 Psychiatry Residency, Community Memorial Healthcare, Ventura, California, United States of America; 2 Department of Psychiatry and Biobehavioral Sciences, David Geffen School of Medicine at UCLA, Los Angeles, California, United States of America; PLOS: Public Library of Science, UNITED KINGDOM OF GREAT BRITAIN AND NORTHERN IRELAND

## Abstract

One of the most concerning pain conditions is Complex Regional Pain Syndrome (CRPS), a nervous system disorder that may occur after a trauma, surgery, medical procedure, or prolonged immobilization. Its primary symptom is extreme and constant burning or freezing pain at the site of the inciting injury, often out of proportion to, and lasting longer than, the injury itself. A psychiatric condition that needs to be differentiated from CRPS is Somatic Symptom Disorder (SSD), which was added to the Diagnostic and Statistical Manual of Psychiatric Disorders, Fifth Edition (DSM-5) in 2013. It requires only a single physical (somatic) symptom (there may be more), but, equally important, the patient also must have thoughts, feelings, or behaviors that are clearly excessive relative to their physical symptom(s). Differentiating between CRPS and SSD can present a difficult diagnostic challenge but is necessary to improve the well-being of those affected. Both occur in inpatient and outpatient settings. For CRPS, diagnosis relies on assessment according to specific clinical criteria, because there are no definitive diagnostic tests. SSD is characterized by excessive preoccupation with somatic symptom(s), including pain, and there also are no definitive diagnostic tests. Because both conditions share the characteristic of distressing somatic symptom(s), and both have important psychological components, differential diagnosis often requires extensive investigation. To illustrate their diagnostic complexities, in addition to the existing literature, we use case examples of CRPS, SSD, and a combination of both. These cases highlight the need for multidisciplinary collaboration in evaluating and managing both disorders, in order to address both the physiological and the psychological components. Consultation-liaison psychiatrists, in particular, have requisite training in both domains and can have a crucial collaborative role, acknowledging both the extent of physical pain and addressing psychological dimensions, including anxiety, depression, and the magnification of underlying physical complaints.

## 1. Introduction

In general practice, physicians and allied clinical staff commonly encounter a variety of psychiatric disorders, ranging from mood disorders to acute psychosis. Psychosomatic disorders also are encountered; for instance, somatic symptom disorder (SSD). SSD has an estimated frequency of 13% in the general population, with no apparent sex differences in adults [1]. Affected individuals often seek evaluation for a variety of somatic complaints including fatigue, weakness, subjective fever/chills, abnormal sensory experiences, chronic pain, and physical disability. Multiple symptoms can trigger extensive evaluations, including laboratory tests and diagnostic imaging. In the majority of cases, however, because of multiple patient-related and clinician-related diagnostic barriers [2], the work-up does not yield sufficient evidence to identify a clear cause of the patient’s complaints. Clinicians therefore conclude that the patient’s somatic complaints are more likely psychiatric in nature. The patient is then referred for psychiatric evaluation, which can be helpful in elucidating any underlying psychiatric disorders that might explain the patient’s complaints.

There also are patients, however, with seemingly unexplained symptoms that may be erroneously attributed to a psychiatric origin. Patients with complex regional pain syndrome (CRPS), in particular, are at a greater risk of being inappropriately labeled as having psychosomatic symptoms, which can result in inadequate treatment of their underlying medical condition, thus having an impact on their well-being. In this Essay we describe CRPS and contrast its symptoms and signs with somatic symptom disorder (SSD), the most common somatoform (psychosomatic) syndrome in order to inform management approaches.

CRPS is a nervous system disorder that can occur after a trauma, surgery, medical procedure, or prolonged immobilization. It is characterized by pain that lasts longer and is more severe than expected for the original tissue damage. The primary presenting symptom of CRPS is extreme and constant burning or freezing pain, often out of proportion to the inciting injury. Additional manifestations of CRPS include autonomic changes (e.g., sweating, circulation abnormalities), motor changes (e.g., weakness, muscle spasms), and trophic changes (e.g., skin and/or bone atrophy, hair loss, joint contractures). There are two types of CRPS: In CRPS type I (also known as reflex sympathetic dystrophy), there are no discernible, actual nerve injuries or lesions. CRPS type I comprises about 90% of cases [3]. In CRPS type II (formerly known as causalgia), there is identifiable evidence of nerve damage [4].

CRPS has had an evolving and, at times, controversial history in medicine. An early description was by Ambroise Paré, a pioneer surgeon, who treated King Charles IX of France [5]. Based on the King’s symptoms—severe pain and other neurological disturbances in the affected limb—Paré laid the foundation for understanding how trauma could lead to lasting pain and sensory disturbances, even without apparent tissue damage. Paré also described a related neurological disorder, phantom limb pain, in which amputees experience pain or sensation in limbs that no longer exist. These insights were the early precursors to the recognition of CRPS as a distinct clinical entity.

In 1813, British surgeon Alexander Denmark [6] described a soldier with persistent, burning pain following a gunshot wound, along with skin color changes and temperature fluctuations, which indicated that the condition was not simply related to nerve damage but had a more complex etiology. These observations were promulgated by the American physician Silas Weir Mitchell [7–9], who, after treating soldiers with nerve injury during the American Civil War, noted these soldiers experienced not only intense pain but also swelling, changes in skin color, and altered sensations in the injured limbs. Mitchell coined the term “causalgia” to describe this condition, observing that causalgia could occur even without visible nerve damage, and that the associated pain might be related to central, peripheral, and autonomic nervous system dysfunction, a concept that is a defining feature of CRPS.

In 1900, Paul Sudeck [10] described “Sudeck’s atrophy”–the bone and muscle atrophy that can follow soft tissue injury, also part of CRPS. Later, René Leriche, Jules Tinel, and others [11], including James Evans [12], linked CRPS to sympathetic nervous system dysfunction. Evans coined the term “reflex sympathetic dystrophy” (RSD), emphasizing post-injury dysfunction of the sympathetic autonomic nervous system, which regulates, *inter alia*, blood flow, temperature, and sweating [12]. The term, RSD, underscored the neurovascular nature of CRPS, which guided subsequent research into its underlying mechanisms.

In the 1950s, John Bonica [13], a leading figure in pain management, further advanced the clinical understanding of CRPS by characterizing its progression through acute, dystrophic, and atrophic stages. Bonica’s work with RSD helped establish pain management as a medical specialty and led to the founding in 1973 of the International Association for the Study of Pain (IASP). IASP held consensus conferences in 1988 and 1993 to refine the diagnostic criteria for CRPS, leading to the renaming of RSD to CRPS, thus reflecting its complex and diverse nature with pain as its central feature [14].

There are three stages of CRPS: acute, subacute (dystrophic), and chronic (atrophic) [15]. The acute stage lasts for about three months, during which patients usually have burning pain, hyperesthesia (increased sensitivity to touch) and/or allodynia (pain from unusual stimuli), swelling, skin redness, increased sweating, and decreased range of motion. The subacute stage lasts from three to 12 months and is associated with continuing severe pain, swelling, skin dryness, and paleness or bluish coloration of the skin. After 12 months, CRPS is considered to be chronic and can last for years or even become permanent. In the chronic stage, pain may continue to be severe, or it may subside, and the patient’s skin is usually dry, shiny, and cool to the touch.

Early and late stage CRPS show different functional brain activity [16], and each stage has its own psychological challenges [17]: In the acute stage, there is fear and anxiety owing to the sudden onset of severe pain and worry about long-term effects. In the subacute stage, the persistence of pain can lead to a sense of helplessness and social withdrawal, as functional limitations increase. In the chronic stage, anxiety and depression can increase as physical changes develop in the affected limb, and secondary psychological issues such as post-traumatic stress disorder may emerge, particularly if the CRPS was caused by a traumatic injury.

The onset of CRPS in adults most frequently varies between 37 and 70 years of age; its occurrence is rarer in youth. It occurs 3 to 4 times more frequently in women [18], and the reported incidence is quite variable [19]. CRPS is considered to be multifactorial, with potential contributions from inflammatory, immunological, autonomic, and genetic factors. Risk factors are reported to include asthma, use of angiotensin-converting enzyme-inhibiting medications, menopause, osteoporosis, history of migraine, and cigarette smoking [19], but it is not known how these factors contribute to the development of the condition. There also may be central and peripheral sensitization, which occur when a part of the body has been under painful stimulus for a period of time, after which any stimulus to the affected area produces similar or worse pain.

There is no diagnostic test that reliably identifies CRPS, and as such, it remains a diagnosis of exclusion and is best identified with the Budapest criteria [20]. These include continuing pain disproportionate to an inciting event, and at least one symptom from three of four categories: sensory (e.g. pain on light touch or abnormal sensitivity to pain), vasomotor (e.g. changes in skin color or temperature), sweating (e.g. abnormal sweating or dryness), and motor/trophic (e.g. movement impairment or changes in hair/nail growth). There also must be at least one finding on physical examination to substantiate the reported symptoms in two of these categories. Finally, the condition must not be better explained by another diagnosis.

The Budapest criteria have a reported sensitivity of 0.99 and specificity of 0.68 [21]. The high sensitivity implies that positive cases will almost always be detected, but the rather low specificity implies that true negative cases may be incorrectly identified as CRPS. Thus, other medical diagnoses must be considered and need to be ruled out; examples include small and large fiber sensorimotor neuropathy, local infection, vascular insufficiency or inflammation, lymphedema, deep vein thrombosis, and Reynaud’s phenomenon [22].

Somatic symptom disorder (SSD) is a psychosomatic disorder that was added to the Diagnostic and Statistical Manual of Mental Disorders, Fifth Edition (DSM-5) in 2013 [23]. It replaces several DSM-IV Somatoform Disorder categories (see below). Diagnostic criteria include 1) one or more somatic symptoms that are distressing or that disrupt the individual’s daily life, 2) excessive thoughts, feelings, or behaviors related to the somatic symptoms or associated health concerns (exaggerated thinking about the seriousness of one’s symptoms, persistently high anxiety about one’s symptoms, or excessive time/energy spent on health concerns), and 3) persistence of being symptomatic with at least one symptom (typically greater than six months). Additional specifiers include a) with predominant pain, b) persistent (severe symptoms, marked impairment, long duration), and c) severity (mild, moderate, severe). The severity threshold for diagnosis is high: SSD is “associated with marked impairment of health status and high psychological distress…Health status is particularly impaired in the presence of multiple or severe symptoms.” [23].

Just as one must consider a variety of conditions before making a diagnosis of CRPS, one must also consider a variety of medical conditions before diagnosing SSD or other psychosomatic condition. “The presence of somatic symptoms of unclear etiology is not in itself sufficient to make the diagnosis of [SSD]…Conversely, the presence of somatic symptoms of an established medical condition…does not exclude the diagnosis of [SSD] if the criteria are otherwise met.” [23]. This can be a complicated undertaking, because there are a number of similarities in diagnostic criteria between CRPS and SSD, as detailed below. It is important to remember that in SSD, “the individual’s suffering is authentic, whether or not it is medically explained” [23]. This is equally true for CRPS.

In order to highlight the differences between these conditions and manage them accordingly, it is important to consider the changes in the diagnostic criteria for SSD (formerly somatoform disorder). In the previous edition of the Diagnostic and Statistical Manual of Mental Disorders (DSM-IV), the criteria for somatoform disorder included the patient’s experiencing at least four different pain symptoms from a combination of several systems; e.g., two gastrointestinal, one sexual, and one pseudo-neurologic type pain [24]. With the advent of DSM-5, however, the new diagnosis of SSD was added, and the diagnostic criteria were altered to require only a single somatic symptom [25]. Of importance, in SSD increased emphasis is placed on excessive thoughts, feelings, and behaviors related to the somatic symptom(s), rather than on the presence of multiple, medically unexplained symptoms and/or pain, as in DSM-IV.

The DSM-5 diagnosis of SSD also replaced several previous diagnoses, including somatization disorder, pain disorder, and undifferentiated somatoform disorder [25], and it helped address several criticisms of the diagnosis of somatoform disorder, including the questionable importance of multiple, unexplained symptoms and/or pain. For example, SSD has been considered an advance over the previous DSM-IV pain disorder, because SSD removes the requirement that symptoms must be medically unexplained, and it adds the criterion of major and excessive psychological distress [25].

On the other hand, there are concerns about the diagnosis of SSD: In particular, it can be perceived as overly inclusive, leading to a higher probability of medical conditions, including chronic pain conditions, being misdiagnosed as mental illness [25]. Frances [26] cited an American Psychiatric Association meeting presentation of field studies indicating that the diagnosis of SSD had a false positive rate of 7% among healthy people in the general population, and that 15% of patients with cancer, 15% of patients with heart disease, and 25% of patients with irritable bowel syndrome and chronic widespread pain would qualify as having SSD. Therefore, although the aim of altering the diagnostic criteria for somatoform disorder (DSM-IV) to those of SSD (DSM-5) was to simplify and clarify the diagnostic procedure for use in primary care [27], the changes likely served to decrease diagnostic clarity in cases of chronic pain disorder such as CRPS.

To provide guidance for differentiating CRPS from somatic symptom disorder, the most common “psychosomatic” syndrome, we present three reports of patients with complex symptomatology who ultimately were diagnosed with CRPS, SSD, or both.

## 2. Case reports

### 2a. Complex Regional Pain Syndrome (CRPS)

Araki et al. [28] reported a 25-year-old woman with persistent pain and distinctive skin abnormalities on her left hand, which developed following a sprain of her left wrist a year prior. She experienced escalating burning pain, swelling, cold intolerance, numbness, and muscle weakness extending throughout her left hand. Physical therapy had yielded no improvement. Examination of her left hand revealed pale, swollen, and tense skin, with hair loss, decreased sweating, and a shiny appearance resembling scleroderma of the fingers. The dorsal skin over the fingers was tense and unable to be pinched. There was mild flexion contracture of the third, fourth, and fifth fingers.

Routine laboratory tests showed no abnormalities, while neurological examination revealed increased pain sensitivity, abnormal skin sensations including to touch, and muscle weakness in the left forearm and hand. Nerve conduction studies were within normal limits. In addition, radiographic examination revealed a significant loss of bone calcium in the third, fourth, and fifth fingers of the left hand, and thermography revealed a 2.6°C lower temperature of the top of the left hand compared to the right. Similarly presenting autoimmune diseases were ruled out, based on the unilateral involvement, neurological symptoms, and absence of autoantibodies. In view of the patient’s trauma history, symptoms, findings on examination, and laboratory and other studies, a diagnosis of CRPS Type I was made.

In this case, treatment involved stellate ganglion (local sympathetic nervous system) blockade and infrared therapy. It resulted in temporary pain relief, decreased pain sensitivity, and improved range of motion. Despite 10 months of continuous treatment, however, the pain and swelling progressed and spread to the patient’s right hand.

This case highlights the challenging clinical course of CRPS I, characterized by persistent pain and skin changes; it underscores the limited long-term efficacy of conventional therapeutic approaches; and it emphasizes the need for multidisciplinary interventions and further research to enhance treatment strategies and improve well-being.

### 2b. Somatic Symptom Disorder (SSD)

Dunphy et al. [24] reported a 31-year-old woman who presented to the emergency department with recurrent headaches, double vision, and left-sided abdominal and leg pain over the previous year. She described the abdominal pain as "sharp, shooting, and stabbing" and had one episode of nausea and vomiting. Her past medical history was complex, with multiple emergency department visits and a myriad of symptoms, raising concerns about possible underlying causes. However, lumbar puncture, computed tomography (CT) scans, and ultrasounds were consistently within normal limits. Her persistent, distressing symptoms and the absence of abnormalities on examinations prompted a multidisciplinary team to perform a collaborative assessment. Psychiatric evaluation revealed a history of chronic depression and “an emotionally unstable personality disorder,” elucidating a potential interaction between the patient’s mental health and her physical symptoms. Her continuing demand for additional studies and excessively dire thoughts concerning her abdominal pain suggested the possibility of SSD. Her psychiatric assessment was key in confirming the diagnosis of SSD and in highlighting the major psychosocial dimensions of her condition. Post-diagnosis, the patient was referred to the “medically unexplained symptoms clinic,” where she received several weeks of supportive care and “extensive investigations,” after which she was discharged home uneventfully. No further follow up information was provided.

This case underscores the challenges in diagnosing SSD, especially when pain predominates. Nuanced, multidisciplinary collaboration among clinicians is key to effective management of patients presenting with somatic symptoms without discernible organic causes, as well as patients having both psychosomatic symptoms and organic pathology [29, 30].

### 2c. CRPS + SSD

Sehdev et al. [31] reported a 16-year-old boy with Crohn’s disease (inflammatory bowel disease), in remission with anti-tumor necrosis factor (TNF) treatment (adalimumab), who presented with acute-on-chronic bilateral lower extremity pain, initially attributed to an overuse injury post-Junior Olympics participation. He also complained of peripheral numbness, tingling, burning, cooling sensation, and blue/red skin discoloration to his right lower extremity. His symptoms progressed, leading to loss of function and muscle weakness. Neurology consultation was requested, owing to possible foot drop secondary to peripheral demyelination from the adalimumab treatment for his Crohn’s disease. Extensive evaluations included electromyography and a trial of intravenous immunoglobulin and gabapentin to target possible demyelination. Despite initial improvement with gabapentin, following a left ankle injury from a biking accident, the patient’s symptoms escalated, mirroring the symptoms previously affecting his right leg. He also developed skin ulcers and streaks of hair loss on both legs. These lesions further complicated diagnostic considerations, prompting a multidisciplinary approach that included pediatric gastroenterology, dermatology, immunology, rheumatology, neurology, and pain medicine. Skin biopsy revealed nonspecific, reactive hypercellular prejunctional fibrosis (scarring with an excess of cells), which did not point to a clear cause for his symptoms.

As the patient’s medical examinations progressed, his psychological state became a significant factor, marked by intense anxiety and catastrophic thoughts related to both his gastrointestinal symptoms and his pain. He reported daily stomach aches associated with anxiety, despite testing negative for Crohn’s disease flares. His score on the Wong-Baker Faces Pain Rating Scale [32, 33] was 10/10, reflecting the severity of his distress. He then was evaluated by a pain psychologist whose diagnoses included SSD and anxiety disorder, with "massive, paralyzing" anxiety about gastrointestinal and CRPS symptoms. Following a diagnosis of both CRPS and SSD, the patient was treated with weekly, in-person physical therapy (PT) and cognitive-behavioral therapy (CBT). With this coordinated intervention, his excessive sensitivity to pain, immobility, and skin lesions gradually resolved over the next four months. Objective assessments with the Lower Extremity Functional Scale [34, 35] and the Central Sensitization Inventory [36, 37] showed a substantial improvement in functional state to a level indicating no impairment and a transition from mild to subclinical sensitization.

This case of CRPS with later, superimposed SSD underscores the interplay of physiological and psychological elements in chronic pain that necessitated a broad and tailored approach addressing this young patient’s multifaceted illness experience [38]. In children and adolescents, transitions between developmental stages; e.g., the onset of puberty, also pose special concerns that may require adjustment of ongoing treatment.

## 3. Discussion

**Fig 1:**
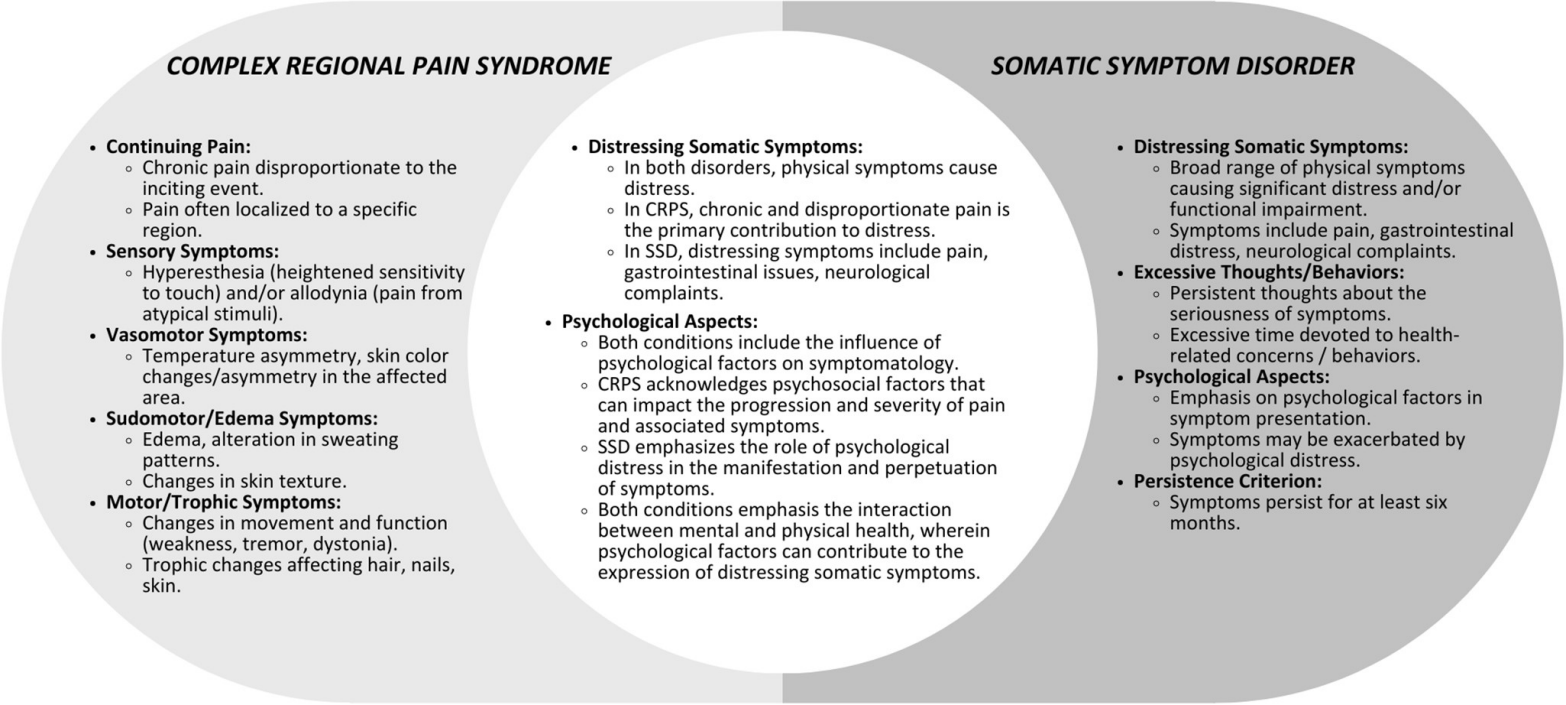
Symptom overlap between CRPS and SSD.

Understanding and effectively treating persistent physical symptoms, especially chronic pain, can be daunting for both mental health and medical professionals [2]. We have illustrated the complexity and challenges in differentiating CRPS from SSD and the necessity for a comprehensive understanding of the physiological and psychological dimensions of both conditions. Clinicians must be aware of the potential for misdiagnosis of patients who present with unexplained somatic symptoms, as is the case for many patients with CRPS. In practice, this can be a difficult undertaking, as exemplified by the three cases presented above and by Fig 1, which illustrates the considerable symptom overlap between the two disorders.

The multiple pathological features of CRPS result in a variety of clinical presentations with varying degrees of severity. The absence of a definitive diagnostic test places a heavy reliance on the Budapest criteria [20, 21], a set of clinical guidelines that, while highly sensitive, lack specificity. The clinical heterogeneity of CRPS, encompassing both type I and type II variants, necessitates an understanding of the nuances within each subtype and underscores the importance of a comprehensive evaluation of both physical symptoms and psychological factors that can contribute to the patient’s experience. Moreover, CRPS progresses through distinct stages—acute, subacute, and chronic—each presenting its own set of challenges in diagnosis and management [15, 17].

**Fig 2:**
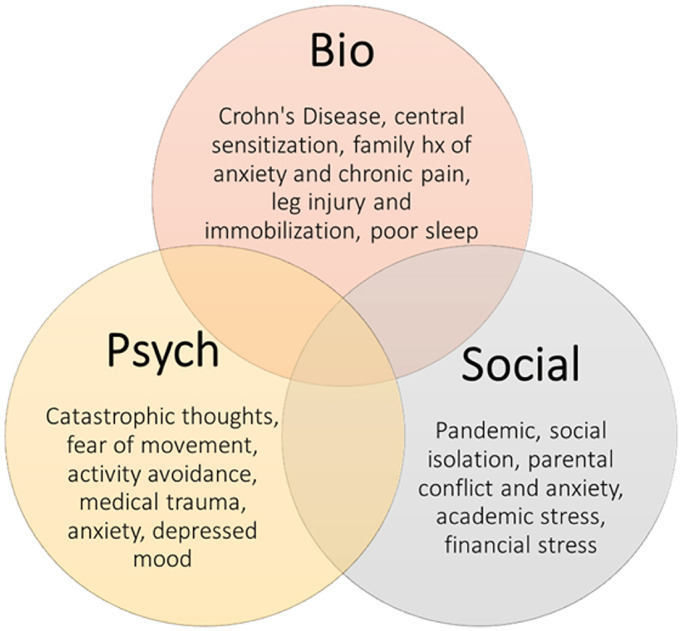
Biopsychosocial approach to a patient with both CRPS and SSD [31]; reproduced with permission from the authors.

The introduction of SSD in DSM-5 also poses diagnostic challenges [1, 29]. The changes from somatoform disorder in DSM-IV were made in order to reduce the misdiagnosis of medical conditions (e.g., CRPS, fibromyalgia, lupus) as somatoform disorders, but the effectiveness of these changes remains unclear. The DSM-5 emphasis on a single somatic symptom, vs. multiple symptoms in DSM-IV, may foster over-diagnosis of SSD in patients with underlying medical conditions (e.g., cancer, heart disease, chronic pain syndromes). Understanding the nuances of presentation of both CRPS and SSD should improve diagnostic accuracy for patients with unexplained pain and somatic symptoms. In particular, framing complex cases in terms of a biopsychosocial model (Fig 2), as was done for case 3, above, can lead to better understanding of their complexity. In both conditions, it is important to appreciate the intricate interplay between mental and physical health and how psychological factors can contribute to distressing somatic symptoms in order to provide holistic, efficacious treatment [39, 40]. Fortunately, helpful instruments such as the Individual Challenge Inventory Tool are being developed to aid clinicians in this endeavor [41].

The integration of psychiatric evaluation with consultations from neurology, gastroenterology, dermatology, immunology, rheumatology, and pain medicine can be crucial for accurate diagnosis and management of patients with both CRPS and SSD. Given that there is no clear underlying pathophysiology of CRPS, treatment is multifaceted and requires a plan tailored to the needs of each patient [42]. With this in mind, it is crucial to consider both pharmacological and non-pharmacological interventions in managing CRPS. Although psychiatric comorbidity may not be significantly higher in CRPS (29%) vs non-CRPS (21%) patients [43], there can be a strong association between the chronic pain and disability of CRPS and psychiatric issues, including anxiety and depression. Therefore, both psychotropic medication and psychotherapeutic intervention may be indicated in the patient’s overall treatment.

Given the complexities of differential diagnosis, as emphasized above, another consideration is that clinicians may become frustrated with an array of confusing symptoms in both CRPS and SSD and step away from a therapeutic stance that requires considerable patience. For example, a middle-aged woman with “unspecified pain syndrome of the nose” had multiple specialist evaluations and treatments and reportedly was told by her psychiatrist that her thoughts were “foolish.” This led the patient to feel hopeless and responsible for her unrelenting pain, culminating in a suicide attempt [44]. The importance of maintaining the therapeutic alliance and being attentive to its components (e.g., transference and countertransference) in such complex cases is clear.

## 4. Conclusion

The diagnosis of both CRPS and SSD requires a nuanced and comprehensive approach. Hill et al. [45] challenged the traditional psychogenic model of CRPS, proposing a more intricate understanding of the condition by examining the relationship between CPRS and SSD. CRPS often coexists with conditions such as depression, anxiety, and insomnia, and it is crucial to recognize that this comorbidity is not indicative of a psychopathological origin of the entirety of the patient’s symptoms [43, 46]; it is essential for health professionals to avoid disregarding CRPS symptoms as driven solely by emotional distress. Attributing CPRS solely to mental health issues can hinder access to crucial treatment interventions, including pharmacotherapy and physical rehabilitation, and can set off a chain of negative psychosocial events. Therefore, there is a pressing need for education among medical and mental health professionals about CRPS symptoms, potential alternative diagnoses, and comorbid conditions, including SSD and other psychiatric disorders, to prevent such misjudgments and ensure appropriate care for affected individuals.

Brinkers et al. [47] emphasize the role of consultation-liaison psychiatrists in addressing the psychiatric dimension of CRPS. Their findings also highlight the coexistence of psychiatric disorders with CRPS, emphasizing the importance of collaboration among healthcare professionals for accurate diagnosis and tailored interventions, as we have noted above. Pain perception is a complex phenomenon influenced by psychological and emotional factors, and the interplay between psychiatric conditions and pain in CRPS patients necessitates a holistic approach to diagnosis and management.

Regarding SSD, primary care practices often represent the first medical encounters for patients with SSD. As noted earlier, the diagnosis of SSD has shown a false positive rate of 7% even among healthy people in the general population [26], so that both underdiagnosis and overdiagnosis of SSD need to be minimized. Fortunately, increased attention is being given to the care of patients with SSD in primary care [29], aided by the development of evaluation instruments like the Individual Challenge Inventory Tool [41].

Incorporating insights from the studies and case reports reviewed above ensures that pain is viewed not merely as a somatic symptom but as a complex interplay of physiological and psychological factors. By incorporating consultation-liaison psychiatrists and their similarly trained colleagues into multidisciplinary teams, practitioners can navigate the complexities of both CRPS and SSD, ensuring a holistic understanding of these conditions and facilitating more personalized and effective treatment plans.
